# The value of platelet/lymphocyte ratio in young patient with acute ischemic stroke

**DOI:** 10.2478/abm-2023-0067

**Published:** 2023-12-28

**Authors:** Editorial Office of Asian Biomedicine

**Affiliations:** Faculty of Medicine, Chulalongkorn University, Bangkok 10330, Thailand

The incidence of arterial ischemic stroke (AIS) in young adults (aged below 55 years) has been increasing in recent decades despite conditions such as hypertension, smoking, diabetes, and hypercholesterolemia not being as common in young adults as in the elderly [1]. Young people have different risks for stroke as compared with the elderly. The risks include age and sex-specific factors such as pregnancy/puerperium and oral contraceptive use. Additionally, young people tend to be involved in behavioral risks such as excess alcohol consumption, smoking, and the use of illicit drugs such as cocaine. Other conditions that contribute to ischemic stroke in young adults are cardiac (e.g., congenital heart disease, atrial fibrillation, and patent foramen ovale), hematologic (such as anti-phospholipid syndrome), and vascular (e.g., Takayasu disease) [2]. In some populations, sickle cell disease can result in the development of stroke after acute clinical and subclinical infections [3]. An increased incidence of stroke and transient ischemic attack has been observed in patients with rheumatoid arthritis and ankylosing spondylitis [4]. Young stroke survivors also often have other adverse long term outcomes, including epilepsy, pain, cognitive problems, and depression [4].

The natural histories of young patients with ischemic stroke vary. Attempts have recently been made to predict the prognoses of these patients [5]. Some studies maintain that “high-sensitivity C reactive protein, neutrophil/lymphocyte ratio and platelet/lymphocyte ratio (PLR) were strongly correlated with the severity of stroke” [5]. Additionally, a meta-analysis suggested that a high PLR was significantly and negatively associated with radiological bleeding mortality and 90 d functional outcomes [6]. However, a different meta-analysis indicates that PLR may not be a useful prognostic marker to predict functional outcomes after acute ischemic stroke [7].

In this issue, Wen et al. [8] report that a high PLR value may predict the possibility of hemorrhagic transformation in young patients with acute ischemic stroke, with a sensitivity and a specificity of 0.806 and 0.674, respectively. Because of the contradictory results among different studies and since PLR values can be readily available at low cost, there is a need to design a good randomized controlled study to provide a definitive answer to the potential role of PLR in AIS.
